# Social determinants of health and disparities in prenatal care utilization during the Great Recession period 2005-2010

**DOI:** 10.1186/s12884-019-2486-1

**Published:** 2019-10-29

**Authors:** Erin L. Blakeney, Jerald R. Herting, Betty Bekemeier, Brenda K. Zierler

**Affiliations:** 1 Department of Biobehavioral Nursing and Health Informatics, Center for Health Sciences Interprofessional Education, Research, and Practice (CHSIE), Seattle, USA; 2 Department of Sociology, University of Washington, Box 353340, Seattle, WA 98195 USA; 3 School of Nursing, University of Washington, UW Health Sciences Building, Box 357266, Seattle, WA 98195 USA

**Keywords:** WIC, Prenatal care utilization, Great recession, Disparities, Partisan voting patterns, Social behavior

## Abstract

**Background:**

Early, regular prenatal care utilization is an important strategy for improving maternal and infant health outcomes. The purpose of this study is to better understand contributing factors to disparate prenatal care utilization outcomes among women of different racial/ethnic and social status groups before, during, and after the Great Recession (December 2007–June 2009).

**Methods:**

Data from 678,235 Washington (WA) and Florida (FL) birth certificates were linked to community and state characteristic data to carry out cross-sectional pooled time series analyses with institutional review board approval for human subjects’ research. Predictors of on-time as compared to late or non-entry to prenatal care utilization (late/no prenatal care utilization) were identified and compared among pregnant women. Also explored was a simulated triadic relationship among time (within recession-related periods), social characteristics, and prenatal care utilization by clustering individual predictors into three scenarios representing low, average, and high degrees of social disadvantage.

**Results:**

Individual and community indicators of need (e.g., maternal Medicaid enrollment, unemployment rate) increased during the Recession. Associations between late/no prenatal care utilization and individual-level characteristics (including disparate associations among race/ethnicity groups) did not shift greatly with young maternal age and having less than a high school education remaining the largest contributors to late/no prenatal care utilization. In contrast, individual maternal enrollment in a supplemental nutrition program for women, infants, and children (WIC) exhibited a protective association against late/no prenatal care utilization. The magnitude of association between community-level partisan voting patterns and expenditures on some maternal child health programs increased in non-beneficial directions. Simulated scenarios show a high combined impact on prenatal care utilization among women who have multiple disadvantages.

**Conclusions:**

Our findings provide a compelling picture of the important roles that individual characteristics—particularly low education and young age—play in late/no prenatal care utilization among pregnant women. Targeted outreach to individuals with high disadvantage characteristics, particularly those with multiple disadvantages, may help to increase first trimester entry to utilization of prenatal care. Finally, WIC may have played a valuable role in reducing late/no prenatal care utilization, and its effectiveness during the Great Recession as a policy-based approach to reducing late/no prenatal care utilization should be further explored.

## Background

During the Great Recession in the United States (U.S.), indicators of need—such as the percent of children in poverty, unemployment rates, and consumer distress—increased [1–3]. Historically, Black and Hispanic populations have had higher rates of unemployment compared to Whites, and during and after the Great Recession these disparate rates were maintained [4]. All ethnic groups experienced increases in unemployment during the Great Recession, but Blacks continued to have the highest unemployment rates, Whites had the lowest, and Hispanics fell between the two [4]. At the same time, community-level safety net resources, including many maternal and child health programs provided by local community health departments (LHDs), experienced cuts which may have contributed to increased difficulties among pregnant women in accessing prenatal care —particularly during the earlier phases of the Great Recession and before federal stimulus funds became available [5–7].

Early (within the first trimester) and regular PNC is known to be an important strategy for improving health outcomes for mothers and infants [8, 9]. Improved birth weight and decreased risk of preterm delivery are two of the most significant benefits of early and ongoing utilization of prenatal care [9, 10]. Infants born to women who do not receive prenatal care are three times more likely to have a low birth weight and five times more likely to die than infants born to mothers who receive prenatal care [11, 12]. Improved infant health outcomes associated with early utilization of prenatal care have both quality of life and cost implications. Average medical costs for a premature or low birth weight infant during the first year of life are about $55,393, whereas annual costs for a newborn without complications averages $5085 [13].

Racial/ethnic disparities in timing of entry to prenatal care utilization are well documented and persistent in the U.S.—despite improvements in recent years and national attention to disparity elimination as a primary goal of the U.S. Department of Health and Human Services (DHHS) Healthy People program [8, 14]. Disparities are widely recognized to be complex and multi-faceted at many levels. Their existence ranges from differences that are apparent at the individual level to health outcomes that represent macro-social differences in political ideologies and wealth distribution [15, 16]. Previous research has found that persistent disparities associated with prenatal care utilization are predominantly related to social determinants of health including social circumstances, access to medical care, and behavioral patterns [17].

Changes in individual and community resources during the Great Recession raise questions as to whether existing disparity relationships—defined as differences in rates of early (first trimester) as opposed to late/no entry to prenatal care utilization among different socioeconomic groups—might also be influenced. During a recession in the early 1980’s, Fisher, LoGerfo, and Daling [18] found increases in late entry to prenatal care utilization in Washington (WA) State. In that study, the authors found specific increases among those who resided in low income census tracts (compared to high); however they did not explore differential increases among race/ethnicity groups [18]. Using established methods for analyzing disparities [19–21], we also recently found rates of late/no prenatal care utilization increased among some groups during the Great Recession (December 2007–June 2009) in WA and Florida (FL) [22, 23]). For example, our study showed that prior to the Great Recession, 15.3% of White and 20.9% of Black mothers in WA received late or no prenatal care. During the Great Recession, rates of late/no prenatal care utilization increased for both groups—to 17.4 and 28.4%, respectively. The steeper increase among Black mothers yielded a 26.8% increase in disparity in outcomes in relation to White mothers [22]*.* We have also confirmed the presence of prenatal care utilization outcome disparities in WA and FL (the same study population used in this study) among groups defined by race/ethnicity and other social status characteristics (e.g., education, insurance status, age, marital status) [22, 23].

As a result of these preliminary findings and questions, the purpose of this study was to better understand contributing factors to disparate prenatal care utilization outcomes among women of different racial/ethnic & social status groups before, during, & after the Great Recession (December 2007–June 2009). Our hypothesis was that both individual and social characteristics would play important roles in whether and when pregnant women accessed prenatal care (within the first trimester as opposed to after the first trimester or not at all (late/no prenatal care utilization)) and that relative contributions of community/social characteristics would change during the course of the recession as these inputs varied based on the economy and investments in maternal and child health programs.

## Methods

### Study design

In this study we assembled and linked a variety of individual and community-level indicators to better understand factors contributing to disparities in timing of entry to prenatal care utilization among women of different racial/ethnic backgrounds and social status groups before, during, and after the Great Recession (2005–2010). Predictors of entry later than first trimester, including non-entry to prenatal care utilization were identified and compared using a cross-sectional pooled time series design. Particular attention was focused on indicators that may have changed during the Recession, such as unemployment rate, partisan voting patterns, or per capita local health department (LHD) expenditures on a supplemental nutrition program for women, infants, and children (WIC) and other maternal child health programs (Table 1).

**Table 1:** Covariates for regression models

Covariate Level	Covariate Name/Description
Individual	• Race/ethnicity: non-Hispanic White (White), Hispanic White (Hispanic), non-Hispanic Black (Black)^a^ • Maternal age • Marital status (Married/Unmarried) • Mother foreign-born (Yes/No) • Maternal education (Less than HS; HS Diploma or GED; some college not assessed (age < 20 years)) • WIC (maternal WIC enrollment) (Yes/No) • Maternal insurance status (e.g., Medicaid or private insurance).
Community^b^ (at the LHJ level unless otherwise indicated)	• Core Based Statistical Area (CBSA) (metropolitan, micropolitan, or rural) • Community poverty (binary variable, 1 for LHJs with highest percentage (top 1/3) of residents age 0–17 in poverty in each state, 2 for lower number of residents age 0–17 in poverty (non-poor LHJs) • Partisan Voting Patterns: Percent of voters voting Republican (vs. Democrat or Independent) in the 2004 and 2008 presidential elections ^c^ • Gini coefficient (2000 census; measure of income distribution/inequality (0–1), larger number > inequality), measuring levels of income inequality • Per Capita General and Family Practitioner MDs/LHJs (for years 2005, 2008, 2010) • Per capita LHJ unemployment rate^d^
Expenditure^e^	• Total LHD expenditures • WIC expenditures • Family Planning (FP) expenditures • Maternal/Infant/Child/Adolescent (MICA) services expenditures • 2MCH--Combined expenditures for 2 MCH services (FP and MICA)^f^
State	• State-level dummy variables were created for WA and FL to capture any state-level differences.

^a^Race/ethnicity groups were defined using data from two separate variables (maternal race and maternal ethnicity) to create a 3-category combined race/ethnicity variable

^b^Community level covariates were selected based on previous research or for which social determinants of health theories suggest a plausible association to maternal and child health (MCH) outcomes in the context of the Recession [16, 17, 24–32]

^c^The partisan voting patterns measure was intended to act as a proxy for differences in political orientation at the community level as previous research has identified Republican voters as less likely than Democrats to perceive that there are people in the United States who encounter access to care issues and are less likely to support public health reform [27]

^d^Individual unemployment data were not available

^e^LHD-specific per capita expenditure data were included in the preliminary model as the Recession yielded widespread reports of budget cuts to LHDs [7]. Per capita rates were calculated using total LHJ population as a denominator. Differences in fiscal years between WA and FL were reconciled by assigning FL’s FY to the earlier year (e.g., FL FY 2005–2006 associated with WA FY 2005)

^f^MICA [25, 31] represents a composite of similar expenditure categories for WA and FL LHDs that includes comparable intervention activities among LHDs in both states—e.g., home visiting, prenatal health programs

Three recession-related time periods were defined as (1) Baseline Period #0 before the Recession (January 2005–March 2007), (2) Recession Period #1 (December 2007–June 2009—as officially defined by the National Bureau of Economic Research) [6], and (3) Recession Period #2 (July 2009–December 2010) [22]. Per this definition, Recession Period #2 encompasses the months and years after the official Recession Period (#1) during which community-level economic indicators such as unemployment continued to be elevated above baseline (Period #0) levels [4, 33, 34].

In a second analytic phase, we examined a simulated triadic relationship among time, degree of social disadvantage, and late/no entry to prenatal care utilization during three recession-related time periods among pregnant women of different race/ethnicity groups to compare predicted probabilities of late/no prenatal care utilization for three representative scenarios of social disadvantage (“high,” “average,” and “low”). The measures and rationale for each of the scenarios were informed by theory and existing research are further described below in “measures” and Table 2.

**Table 2:** Social Disadvantage Status Characteristic Constellations

Low Disadvantage	Maternal age 30–34 years old, married, not foreign-born, at least some college education, private insurance.
Average	Maternal age 25–29, not foreign-born, at least some college education.
High Disadvantage	Maternal age 15–19 years old, foreign-born, not married, having less than a HS education, without insurance at the time of delivery.

Characteristics representative of an “average” scenario were defined based on majority (modal) population characteristics

Not all possible characteristics included in scenarios (e.g., maternal age 20–24) as they were defined to represent extreme ends of the social advantage/disadvantage spectrum

### Data and study population

De-identified data from all birth certificates from WA and FL for the years 2005–2010 were retrieved through data-sharing agreements with the Departments of Health (DOH) in FL and WA with institutional review board approval from the University of WA and the FL State Department of Health. These states were selected for inclusion as both experienced a tremendous downturn in economic markers during the Great Recession and both had comparable LHD expenditure data available for the study time period [3, 5, 6, 32–34]. The LHD and community data derive from publicly available datasets and have been incorporated into recent maternal and child health-focused studies [26, 32]. Individual birth certificates were linked to county/Local Health Jurisdiction (LHJ)/LHD data using maternal county of residence. All data were cross-sectional and secondary.

The study population consisted of 678,235 individual pregnant women having their first singleton live birth (492,691 in FL; 185,544 in WA) who resided in the 102 LHJs in WA and FL. Non-first time births were excluded to reduce the issues of repeated measures if women had more than one birth during the study period as linking of maternal data between years was not possible. Multiple births were also excluded (only singletons were kept) as multiple births are associated with increased risk of preterm birth, low birth weight, and infant mortality. LHJs follow county lines in FL and in WA, and in WA, three LHJs were comprised from multiple counties. The study was limited to women whose infants had complete birth certificate information on race/ethnicity, maternal county of residence, and timing of entry to prenatal care utilization. For all individual level variables included in this analysis missing-ness was less than 1.0% with the exception of payment source for delivery which was 0.51% in FL and 2.70% in WA (1.11% overall) and maternal WIC utilization which was missing 1.23% of the time in FL and 9.34% of the time in WA (overall missing = 3.48%).

### Measures

Predictors for the main outcome of entering prenatal care during as compared to after the first trimester of pregnancy (or not at all) were examined. To measure this outcome, a binary variable, based on continuous birth certificate data, was created with “0” indicating those who entered prenatal care during the first trimester and “1” indicating those who entered prenatal care after the first trimester of pregnancy, or who did not utilize prenatal care at all. The authors chose to combine late and non-entry to prenatal care utilization to be parsimonious and to focus the analysis on characteristics of women who entered prenatal care during the first trimester care (the widely accepted standard of care) as compared to those who entered late or not at all. Covariates were selected based on conceptual and previous research linking them to maternal and child health outcomes—individual, community and LHD expenditure measures and state dummy variables were included. Table 1 provides a complete list of these covariates and related literature supporting their incorporation.

To facilitate estimation of combined effects of social disadvantage during the second part of the analysis, individual characteristics found to be related to late/no prenatal care utilization were grouped into scenarios representative of low, average, or high social disadvantage (Table 2) [35]. The authors chose to do this as people have multiple identities and risks [16, 17]. While complex, this step helps to capture the additive (cumulative) impacts of relative advantage or disadvantage. Characteristics representative of an “average” scenario were defined based on majority (modal) population characteristics in the study population. Not all possible characteristics included in scenarios (e.g., maternal age 20–24) as they were defined to represent extreme ends of the social advantage/disadvantage spectrum in the United States.

The low social disadvantage scenario was specified with characteristics associated with “best” outcomes in a previous study using similar data [22]*.* In our regression models these groups were the referents. The average disadvantage scenario was defined based on majority/modal population characteristics. Fewer characteristics were defined for the average scenario as there was not a clear majority with regard to marital status and insurance type at the time of delivery. The high disadvantage scenario was defined as those individual-level characteristics most associated with late/no prenatal care utilization. In this scenario, while maternal age < 14 is the age most highly associated with late/no prenatal care utilization, we substituted maternal age 15–19 as it occurs much more frequently and is also associated with increased risk and poor outcomes.

### Analysis

We carried out analyses in two phases (1) regression model specification to identify predictors of late/no entry to prenatal care utilization for each recession-related period; and (2) estimation of predicted probabilities for race/ethnicity groups for the three social disadvantage scenarios (low, average, and high) at Recession Periods #0, #1, and #2.

#### Phase 1: Regression model specification

Using a pooled cross-sectional time series design, multivariate linear probability regression models (LPMs) were estimated to identify which covariates were predictive of late/no prenatal care utilization for the total study population (WA + FL) during Recession Periods #0, #1, and #2. LPMs were chosen to allow for more readily interpretable results of both analytic phases; results from logistic regression models are similar and are provided in Appendix 2 in Table 8. Models were adjusted first for individual, then community, and finally LHD expenditure covariates described above and in Table 1. We conducted all analyses using STATA version 12 [36]. Clustering of individuals within LHJs was addressed using robust standard errors (SEs), correcting for effects of geographically clustered [37] and for the inherent heteroscedasticity in LPMs. Entry to prenatal care utilization by definition occurs at some point during the nine-month course of pregnancy—because of this proximate relationship, no time lags were introduced into the economic data. A value of *P* < .05 was used to establish statistical significance. Model specification included running models with each of the available LHD expenditure variables. Final preferred model selection was informed by comparing results of Akaike Information Criteria (AIC) and Bayesian Information Criteria (BIC) tests for specified models with the lowest AIC/BIC selected [38].

#### Phase 2: Calculation of predicted probabilities for three social disadvantage scenarios

Following regression modeling, in the second analytic phase, we estimated the predicted probability an individual has of late/no prenatal care utilization given a set of fixed characteristics using the post-estimation margins command in Stata [37, 39]. Values for individual covariate characteristics were set for each of the three social disadvantage scenarios—low, average, and high—and predicted probabilities of late/no prenatal care utilization were calculated for White, Black, and Hispanic subpopulations. This approach facilitated practical interpretation of the combined effects of social status characteristics that tend to cluster together along the range of social advantage/disadvantage. In these calculations, non-specified variables were assessed at their actual observed values [37, 39]. Predicted probability of late/no prenatal care utilization was estimated for the total study population as well as for each state by specifying state dummy variables within scenarios.

## Results

### Profile of women who entered PNC late and summary of economic indicators

The characteristics of the study population are presented in Table 3. Women who entered prenatal care late or not at all (compared to those who entered in the first trimester) were younger (twice as likely to be teenagers), less likely to be married, slightly more likely to be foreign-born, and (of those who could have finished high school) almost twice as likely to have less than a high school (HS) education (9.49% vs. 5.11%). They were also nearly twice as likely to be on Medicaid and had a higher rate of WIC utilization.

**Table 3:** Comparison of demographic and perinatal characteristics: study population for FL, WA, total study population, and United States (n/% unless otherwise indicated) by timing of entry to prenatal care utilization (for entire study period)^a,l^

	FL Late Entry *n* = 100,471 (17.79%) *n* (%)	FL Non-Late Entry *n* = 457,510 (82.2%) *n* (%)	WA Late Entry *n* = 40,037 (18.87%) *n* (%)	WA Non-Late Entry *n* = 169,938 (81.12%) *n* (%)	Total Late Entry *n* = 140,508 (18.30%) *n* (%)	Total Non-Late Entry *n* = 627,448 (81.70%) *n* (%)	U.S. (2008)^b^ n = 1,703,921 *(first births unless otherwise indicated)*
Mother’s Age							
Mother’s Age, years (mean, SD)	22.67 (SD 5.91)	25.78 (SD 6.11)	23.46 (SD 5.70)	26.40 (SD 5.97)	22.90(SD = 5.86)t-test: *p* = 0.00	25.95 (SD = 6.08)t-test: p = 0.00	Total births: 25.1(−-)^c^
Number and proportion of Teenage Births (< 19) as component group of total study population^d^	35,443 (35.28)	75,930 (16.60)	11,211 (27.99)	22,762 (13.39)	46,654 (33.20)	98,692 (15.73)	354,897 (20.8)^b^
Mother’s Race/Ethnicity							
Non-Hispanic White	48,306 (15.24)	268,648 (84.76)	22,117 (16.15)	114,856 (83.85)	70,423 (15.51)	383,504 (84.49)	952,478 (55.90)^b^
Hispanic White^e^	16,215 (24.31)	50,483 (75.69)	8231 (27.77)	21,408 (72.23)	24,446 (25.38)	71,891 (74.62)	360,966 (21.18)^b^
Non-Hispanic Black	26,975 (24.75)	82,009 (75.25)	1998 (25.87)	5725 (74.13)	29,205 (24.83)	87,734 (75.17)	244,340 (14.34)^b^
Maternal SES							
Foreign-Born Mother^f^	32,233 (32.08)	135,358 (29.58)	6518 (16.27)	25,177 (14.81)	38,751 (27.58)	160,535 (25.58)	Total births: --(24.00)^g^
Married (Mother)	28,598 (28.47)	241,714 (52.84)	16,860 (42.27)	110,142 (64.95)	45,458 (32.39)	351,856 (56.11)	Total births: 2,523,146 (59.35)^g^
Number and proportion of Births those with less than High School Education as component group of total study population^h^	9795 (9.79)	23,558 (5.17)	3505 (8.75)	8406 (4.94)	13,300 (9.49)	31,964 (5.11)	Total births:--(20.00) ^i^
Medicaid Birth^j^	63,180 (62.88)	181,290 (39.62)	21,392 (53.52)	51,180 (30.22)	84,572 (60.22)	232,470 (37.08)	Total births 1,715,957 (40.08) ^j^
WIC Utilization	65,434 (66.20)	202,041 (44.72)	19,779 (54.55)	52,869 (34.32)	85,213 (63.07)	254,910 (42.08)	Total births: 2,432,006 (57.25) ^k^

^a^All categoric variables were tested with chi-square and found to be significant at *p* < 0.00 level

^b^ source: reference [35]

^c^ source: reference [36]

^d^U.S. data define teens as age 10–19

^e^ this represent ALL Hispanic White ethnicity mothers (except Cuban)

^f^ report cites 2004 data

^g^ source: reference [39]

^h^For age > 20 in WA and FL; unclear of definition related to age in cited material related to U.S. population

^i^ source: reference [40]

^j^ denominator = 4,280,854; source: reference [41]

^k^ infants in 2008 (up to their first birthday); source: reference [42]

^l^ Missing data: This analysis was limited to birth certificates with complete information on race/ethnicity, maternal county of residence, and timing of entry to PNC. For all individual level data missingness was less than 1.0% with the exception of payment source for delivery which was 0.51% in FL and 2.70% in WA (1.11% overall) and maternal WIC utilization which was missing 1.23% of the time in FL and 9.34% of the time in WA (overall missing = 3.48%)

During the study period, unemployment increased dramatically in both states (Table 4). FL unemployment rates more than doubled by Period #1 and then tripled by Period #2 from baseline. In WA, unemployment increased, but not as dramatically—from 5.14% (SD 0.94%) at baseline to 6.61% (SD 2.20%) during Period #1 and to 9.71% (SD 1.54%) during Period #2. WIC enrollments and Medicaid as a proportion of payers also increased in both states, but more in FL than in WA for both indicators. Per capita LHD expenditures varied widely in both states, but mean expenditures had an overall trend toward decreased per capita spending for family planning (FP) and for a composite of maternal/infant/child/adolescent (MICA) service lines [26, 32]. We also combined FP and MICA to create the 2MCH expenditure variable (combined expenditures for two maternal and child health (MCH) services —FP and MICA) in our regression models (Table 1) in both states over the course of the study period. Among LHDs in FL, per capita 2MCH expenditures decreased from $8.79 (SD $5.67) during the baseline period to $8.18 (SD $5.54) during Period #1 and to $7.84 (SD $5.11) during Period #2. In contrast to LHD decreases in 2MCH expenditures, WIC expenditures among LHDs generally increased during the study period in both states—from $4.10 (SD = $1.98) during the baseline period to $4.55 (SD = $2.30) during Period #1 and $5.02 (SD = $2.60) during Period #2.

**Table 4:** Descriptive summary of economic indicators before and during recession periods in WA and FL (Total Study Population)

	FL baseline	FL Period 1	FL Period 2	WA baseline	WA Period 1	WA Period 2	Total baseline	Total Period 1	Total Period 2
Mother Enrolled in WIC while pregnant *n* (%)	94,590 (45.25)	72,677 (49.88)	69,921 (53.21)	24,915 (37.91)	20,706 (38.79)	19,078 (38.78)	119,505 (43.49)	93,383 (46.91)	88,999 (49.28)
Medicaid birthsn (%)	90,375 (42.53)	64,495 (43.76)	62,501 (47.06)	25,769 (34.72)	19,811 (34.45)	18,688 (35.11)	116,144 (40.51)	84,306 (41.14)	81,189 (43.64)
LHD Expenditures: Avg. Total $ (SD)									
Avg. Total (per capita) LHD expenditures	$46.77 (21.64)	$46.16 (22.13)	$46.30 (21.82)	$57.89 (30.21)	$56.29 (29.02)	$54.38 (29.71)	$49.64 (24.63)	$49.00 (24.68)	$48.50 (24.53)
Avg. 2 MCH (per capita) expendituresNote: FL has July start FY	$8.79 (5.67)	$8.18 (5.53)	$7.84 (5.11)	$11.01 (9.06)	$10.46 (9.36)	$9.85 (9.47)	$9.37 (6.79)	$8.82 (6.90)	$8.41 (6.72)
Avg. Family Planning (per capita) expenditures	$3.58 (2.25)	$3.19 (1.80)	$3.08 (1.77)	$2.40 (2.66)	$2.00 (2.48)	$1.72 (2.29)	$3.28 (2.42)	$2.86 (2.08)	$2.69 (2.03)
Avg. MICA (per capita) expenditures	$5.21 (4.44)	$4.99 (4.51)	$4.76 (4.10)	$8.56 (6.75)	$8.42 (7.19)	$8.12 (7.49)	$6.07 (5.34)	$5.95 (5.62)	$5.72 (5.51)
Avg. WIC (per capita) expenditures	$4.64 (1.79)	$5.37 (1.96)	$5.99 (2.13)	$2.57 (1.70)	$ 2.48 (1.70)	$ 2.60 (2.03)	$4.10 (1.98)	$4.55 (2.30)	$5.02 (2.60)
Un-employment rate	3.64%(0.56)	7.39% (2.28)	11.02%(1.41)	5.14%(0.94)	6.61% (2.20)	9.71% (1.54)	4.02% (0.95)	7.17% (2.29)	10.65% (1.56)
Percent voting Republican	2004: 51.97% (10.24)	2008: 47.50% (10.14)	2008: 47.50%(10.14)	2004: 46.80% (10.56)	2008: 41.51% (10.67)	2008: 41.51%(10.67)	2004:50.56%(10.58)	2008:45.86 (28.60)	2008:45.86 (28.60)
Per Capita MDs Family Medicine and General Practitioners	20,050.03 (0.01)	2008 0.03 (0.01)	20,100.03 (0.01)	20,050.04 (0.01)	2008 0.04 (0.01)	2010.0.04 (0.01)	2005 0.03 (0.01)	2008 0.03 (0.01)	2010 0.03 (0.01)

See methods section for explanation of FY: FL has July start FY

Abbreviations (in order of appearance in table from top to bottom): WA: State of Washington; FL: State of Florida; WIC: Special Supplemental Nutrition Program for Women, Infants, and Children; LHD: Local health department; SD: Standard deviation; 2MCH: combined FP and MICA expenditures; FY: Fiscal year; MICA: Maternal, infant, child, and adolescent (service line composite LHD expenditures); MD: medical doctor; GP: general practitioner; FM: family medicine

#### Phase 1: Regression models results within and between periods

Table 5 summarizes the results of all final models (for Recession Periods #0, #1, and #2).. Only minor variations in coefficient magnitudes were found among individual-level categorical characteristics within model steps or across study periods. For example, the difference in probability of late/no prenatal care utilization for Black mothers (compared to the White reference group) was positive during all steps and periods and increased only slightly over time (from 0.032 to 0.037). All individual-level coefficients were positive with the exception of maternal WIC enrollment—which exhibited a relatively stable negative coefficient (− 0.010 to − 0.012). The largest magnitude individual-level predictors were young age (age < 14 and to a lesser degree age 15–19) and having less than a HS education. Those aged 14 years and younger had a 0.259 to 0.262 greater probability of late/no prenatal care utilization compared to the referent group (age 30–34), while those age 15–19 had a 0.087 to 0.097 greater probability of late/no prenatal care utilization than the referent group. Women who had less than a HS education had a 0.061 to 0.084 greater probability of late/no prenatal care utilization compared to women with at least some college. Having Medicaid or being uninsured (self-pay) were also significant positive predictors during both Recession Periods #1 and #2, but not during the Baseline Period.

**Table 5:** Final Late/No Prenatal Care Utilization Linear Regression Models for Baseline, Period 1 and Period 2 (controlled for *102 LHD Clusters)*

	Baseline Period *n* = 270,775			Period 1 *n* = 195,921			Period 2 *n* = 178,254	
	Coef.*	95% C.I.	Coef.	95% CI	Coef.		95% C.I.	
Maternal Race/Ethnicity								
Non-Hispanic White	referent	referent			referent			
Hispanic White	0.020*	0.000–0.039	0.007		−0.010- 0.024	0.009		−0.005- 0.022
Non-Hispanic Black	0.032*	0.020–0.045	0.037*		0.025–0.049	0.037*		0.027–0.046
Age								
< 14 years	0.259*	0.229–0.289	0.261*		0.224–0.297	0.262*		0.201–0.323
15–19 years	0.097*	0.084–0.110	0.091*		0.069–0.112	0.087*		0.075–0.098
20–24 years	0.040*	0.031–0.048	0.050*		0.038–0.060	0.039*		0.031–0.048
25–29 years	0.009*	0.004–0.013	0.011*		0.005–0.018	0.011*		0.006–0.016
30–34 years	referent		referent			referent		
35–39 years	0.002	−0.002-0.007	0.005		− 0.000- 0.010	0.010*		0.001–0.019
40+ years	0.049*	0.032–0.066	0.045*		0.032–0.058	0.029*		0.014–0.044
Marital Status								
Married	referent		referent			referent		
Not Married	0.042*	0.035–0.049	0.042*		0.031–0.053	0.032*		0.024–0.040
Foreign-Born Status								
Not Foreign-Born	referent		referent			referent		
Foreign-Born	0.034*	0.014–0.055	0.028*		0.012–0.044	0.022*		0.009–0.035
Education								
Less than HS education	0.084*	0.064–0.104	0.079*		0.059–0.099	0.061*		0.046–0.076
HS diploma or GED	0.021*	0.012–0.029	0.016*		0.005–0.026	0.020*		0.012–0.029
Some College	referent		referent			referent		
Age < 20; ed. level not assessed	0.056*	0.041–0.070	0.055*		0.036–0.073	0.039*		0.023–0.056
Insurance Payer								
Medicaid	0.100	0.087–0.114	0.113*		0.097–0.129	0.099*		0.085–0.113
Private Insurance	referent		referent			referent		
Self-Pay/ Uninsured	0.155	0.115–0.195	0.173*		0.140–0.205	0.138*		0.102–0.173
Other (Indian Health Service, CHAMPUS, etc.)	0.057	0.017–0.096	0.057*		0.011–0.103	0.070*		0.039–0.100
Unknown	0.038	−0.008- 0.083	0.054		−0.003- 0.111	0.060*		0.016–0.105
WIC Enrollment Status								
Yes WIC	−0.012*	− 0.022- -0.002	− 0.012*		− 0.023- 0.001	−0.010		−0.021 − 0.002
No WIC	referent		referent			referent		
Unemploy-ment Rate	-0.002	−0.018- 0.014	−0.000		−0.003-0.002	− 0.001		− 0.010-0.007
Community Poverty								
Top 1/3 Poor LHJs	−0.031	−0.065 -0.004	− 0.045*		− 0.086- -0.003	−0.056*		−0.096- -0.015
Bottom 2/3 (Non) Poor LHJs	referent		referent			referent		
Median HH Income	7.55E-07	−1.75 E-06- 3.26 E-06	1.53E- 06	1	−8.13E- 07 - 3.88E- 06	8.83E-07		−0.000- 0.000
Core Based Statistical Area								
Metropolitan	referent		referent			referent		
Micropolitan	0.010	−0.021- 0.041	0.003		−0.033- 0.039	0.019		−0.013- 0.050
Rural	−0.016	−0.054 − 0.023	-0.023		− 0.065- 0.020	−0.026		−0.073- 0.022
Gini Coefficient	0.025	−0.442- 0.493	−0.184	0.246	−0.672- 0.304	− 0.152	0.228	− 0.604- 0.301
Percent Republican	0.001*	0.0004-	0.002*		0.0003-	0.002*		0.0005–0.0031
Per Capita MDs (GPs and FM)	−1.498*	−2.959- −0.036	−0.949		−2.213- 0.316	−0.895		−2.265-0.475
State								
Florida	0.056	−0.011- 0.122	0.054		−0.006- 0.114	0.047		−0.013-0.106
Washington	referent		referent			referent		
LHD Per Capita 2MCH Expenditures	0.0012	−0.0007- 0.0030	0.0019*		0.0002–0.0036	0.0025*		0.0009–0.0042
LHD Per Capita WIC Expenditures	−0.0010	−0.0067-0.0046	− 0.0014		− 0.0065- 0.0038	−0.0022		−0.0077- 0.0033

*P < .05 was used to establish statistical significance and is indicated with an asterisk (*)

Abbreviations (in order of appearance in table from top to bottom): Late/No PNC: late (after first trimester) or non-entry to prenatal care; LHD: Local health department; Prob: probability; SE: standard error; Conf.: confidence (for confidence interval); HS: High school; GED: General education diploma; Ed: education; CHAMPUS: Civilian Health and Medical Program of the Uniform Services; WIC: Special Supplemental Nutrition Program for Women, Infants, and Children; LHJ: Local health jurisdiction; HH: household; MD: medical doctor; GP: general practitioner; FM: family medicine;

Three continuous community level variables were significantly associated with late/no prenatal care utilization: (1) per capita MDs (negative coefficient, only significant during the Baseline Period); (2) maternal residence in a high poverty LHJ (negative coefficient, significant during Periods #1 and #2 but not during the Baseline); and (3) percent of LHJ residents voting Republican in a national election (positive coefficient significant during all model steps and time periods—increasing from 0.001 at Baseline to 0.002 during Periods #1 and #2 in the final models). In terms of LHD expenditures, per capita WIC expenditures were negative for late/no prenatal care utilization but not significant at any time period. However, the 2MCH coefficient representing LHD FP and MICA expenditures was positive during each time period (Baseline Period #0 = 0.0012, Period #1 = 0.0019, Period #2 = 0.0025) and significant during Periods #1 and #2. The state dummy variable was not significant.

#### Phase 2: Predicted probability results and comparisons

Results of predicted probability calculations for each of the three social disadvantage scenarios (low, average, high) and race/ethnicity are summarized in Table 6. The predicted values represent the expected probability or expected percentage of individuals (i.e. 0.033 = 3.3%) experiencing late/no care in each group defined by the scenarios in Table 2 and provide a sense of the levels of late/no care experienced by each category of disadvantage and ethnicity/race. Those with combined social characteristics associated with low social disadvantage would be less likely to enter prenatal care late or not at all (range = 0.033 to 0.076) than those with average social status (range = 0.116 to 0.163) for all race/ethnicity groups at all time periods. Those with characteristics representing a high degree of social disadvantage would be the most likely to enter prenatal care late or not at all for all race/ethnicity groups at all time periods (range = 0.379 to 0.482). Differences between race/ethnicity groups within social disadvantage scenarios were much smaller within as compared to between scenarios (the difference between Hispanics and Whites is non-significant and the difference between Blacks and Whites is significant at about 0.03).

**Table 6:** Predicted Probability of Late/No Prenatal Care Utilization in Total Study Population for Low, Average, and High Social Status Characteristics

	Baseline Period		Period 1			Period 2
Total	Prob.*	[95% Conf. Interval]	Prob.*	[95% Conf. Interval]	Prob.*	[95% Conf. Interval]
Low Social Disadvantage Case						
Non-Hispanic White	0.033*	0.024–0.043	0.033*	0.022–0.045	0.039*	0.029–0.049
Hispanic White	0.053*	0.029–0.077	0.040*	0.017–0.063	0.048*	0.030–0.066
Non-Hispanic Black	0.066*	0.048–0.084	0.070*	0.051–0.089	0.076*	0.061–0.091
Average Case						
Non-Hispanic White	0.116*	0.106–0.126	0.126*	0.116–0.136	0.121*	0.111–0.131
Hispanic White	0.136*	0.113–0.159	0.133*	0.114–0.152	0.130*	0.113–0.146
Non-Hispanic Black	0.149*	0.133–0.164	0.163*	0.149–0.178	0.158*	0.145–0.171
High Social Disadvantage Case						
Non-Hispanic White	0.446*	0.377–0.514	0.446*	0.386–0.505	0.379*	0.334–0.423
Hispanic White	0.465*	0.398–0.532	0.452*	0.397–0.508	0.387*	0.344–0.431
Non-Hispanic Black	0.478*	0.412–0.543	0.482*	0.429–0.535	0.415*	0.373–0.458

*P < .05 was used to establish statistical significance and is indicated with an asterisk (*)

Abbreviations (in order of appearance from top to bottom): Late/No PNC: late (after first trimester) or non-entry to prenatal care; Prob: probability; SE: standard error; Conf.: confidence (for confidence interval)

## Discussion

During the Great Recession, we found individual and social characteristics to play important roles in whether and when pregnant women accessed prenatal care. Indicators of need (e.g., maternal Medicaid enrollment, unemployment rate) increased during the Recession in both study states. Young maternal age and having less than a HS education were found to be the largest individual-level contributors to late/no prenatal care utilization among pregnant women in WA and FL during all three recession-related periods. Relative contributions of individual-level predictors were found to exhibit minimal variation across time periods. Simulated scenarios show a high combined impact on prenatal care utilization among women who have multiple disadvantages. Associations between community (particularly percent of the community voting Republican) and LHD expenditure variables and late/no prenatal care utilization revealed variation over time (compared to Baseline Period #0) and increases in the non-beneficial directions. In contrast, individual maternal enrollment in a supplemental nutrition program for women, infants, and children (WIC) exhibited a protective association against late/no prenatal care utilization.

Previous research on the effect of recessions and/or unemployment on maternal and child health outcomes has been used to study a variety of populations as well as outcomes. Among studies that specifically address recessions and MCH outcomes, most found recessions (usually measured by time and/or unemployment rate) to be negatively associated with timing of entry to prenatal care and birth weight and positively associated with infant mortality [18, 43–51]. Race/ethnicity and other individual level characteristics (i.e. maternal education) were rarely taken into consideration in published studies related to past recessions [46, 52]. This paper adds to this body of research by carrying out analyses during the most recent global recession (The Great Recession). Our finding that rates of late/no prenatal care utilization increased during the Great Recession are consistent with previous research. Our explorations of individual and community level contributors to late/no prenatal care utilization extend this research and help set the stage for future research as to whether targeted outreach to individuals with high disadvantage characteristics, particularly those with multiple disadvantages, may help to increase first trimester entry to prenatal care utilization.

In this study, evidence also emerged that WIC may have contributed to reductions in late/no prenatal care utilization over the course of the included recession periods—even in the face of increasing local need. In addition, WIC may have been more effective at reducing late/no prenatal care utilization than the other maternal and child health safety net programs for which we had LHD expenditure data. This finding suggests that the increased WIC enrollment and related increases in local WIC expenditures observed over the course of the Recession may have been particularly beneficial and protective against late/no prenatal care utilization among disadvantaged populations. WIC was the only safety net program for which both individual and community level data were available. It is possible that more nuanced effects among high-need populations targeted by family planning and/or MICA programs with decreasing expenditures were missed; alternatively, results may reflect the general decline in LHD expenditures. WIC may represent a useful policy-based approach to reducing late/no prenatal care utilization and should be further explored.

Regarding LHD expenditures, our findings are consistent with Bekemeier, Yang, Dunbar, Pantazis, and Grembowski [26] who found (using the same LHD expenditure data) that WIC did and 2MCH did not follow changes in local need during the Recession. In our case, LHD expenditures on WIC services were negatively predictive of late/no prenatal care utilization, but not significant at any point. Our findings related to 2MCH were also consistent with Bekemeier et al. [26]. The coefficient size for 2MCH increased over time and was positive rather than negative as might be expected of a maternal and child health program. When considered from the perspective of a $10 increase in per capita maternal and child health expenditures (which would be unlikely as 2MCH budgets generally decreased during the Recession but is a useful example), the probability of late/no prenatal care utilization increased over the course of the study period from 0.01 (0.001 × 10) to 0.03 (0.003 × 10). This is about the same difference in probability observed between Black and White women. During this same time need increased and 2MCH budgets decreased, indicating that the observed increased association may be related to increases in level of need and LHDs stretched to essentially do ‘more with less’ during this study period [53]. Further exploration would be beneficial to understanding this association.

We also identified partisan voting patterns as playing a predictive role in late/no prenatal care utilization. We had included these variables because of prior work by Oakman, et al. [27]. This may an interesting line of inquiry with ongoing shifts in voting patterns and partisan preferences in the United States and beyond.

In the second analytic phase, innovative use of predicted probability methods clearly demonstrate an increased likelihood of late/no prenatal care utilization among women with higher degrees of social disadvantage (Tables 2 and 6). There was little change in these relationships despite changes in need and resources over the course of the Great Recession. While only small changes in coefficient size of race/ethnicity variables were observed in regression modeling and some covariates consistently contributed to a larger degree than others (e.g., education and age were larger contributors than foreign-born status or marital status), the effects of combined social disadvantage become more readily visible when viewed in terms of predicted probability of late/no prenatal care utilization. In these scenarios disparate relationships in prenatal care utilization among Black versus White race/ethnicity groups were maintained, within each level of social disadvantage--with Whites being least likely and Blacks being most likely to enter prenatal care late or not at all. Hispanics consistently fell between Whites and Blacks, though once individual characteristics were controlled for, the difference between Whites and Hispanics was non-significant in these scenarios. These findings demonstrate the cumulative effects of advantage and disadvantage as described by Braveman et al. [35] and Pearlin et al. [25]. Results also suggest that efforts to reduce late/no prenatal care utilization may need to be tailored to best meet the needs of diverse populations based on individual, intermediate, and community characteristics.

### Limitations

There were limitations to this study and some are associated with the review of secondary data (missing or inaccurate). First, while we limited analysis to first-time mothers with singleton births (to reduce issues of repeated measures and increased infant health risks associated with multiple births), generalizability of our results may be limited. In particular, due to the nature of the dataset we were unable to fully address intermediate factors (e.g. distance from a health facility) which undoubtedly play a role in access to prenatal care utilization. Second, we focused the analysis on a binary variable (first trimester entry to prenatal care utilization versus late/no prenatal care utilization) instead of breaking prenatal care utilization into multiple categories. We chose this approach in the interest of parsimony since our analyses focused on differentiating characteristics of women who entered prenatal care utilization during the first trimester care from those who entered late or not at all. This binary approach may have underestimated the impacts of later stages or non-entry to prenatal care utilization and more refined measures of care should be explored in future studies. Third, WA and FL both had heavy economic downturns during the Great Recession and lumping them in the modeling may not have captured key differences or differential impacts within states. To allow for consideration of individual states’ results, we included state-only models for reference in Appendix 3 in Table 9 and Appendix 4 in Table 10. While no significant state-level differences were identified in the models of the total population, demographic differences with WA and FL may have influenced state-level model results. Third, we used 2008 presidential voting data for both Recession Periods #1 and #2, and there may be better measures that would more effectively describe the differences in policy-making than what the partisan voting covariate identifies. Finally, not all WIC expenditures in each state were represented in our models—only those that were expended by LHDs. Some LHJs may have alternative providers of WIC and other maternal and child health services. The non-significant associations that we identified with LHD WIC expenditures may be due in part to this as well as to the fact that WIC is a targeted, need-based program while our study population represented all pregnant women and not only those with need and/or who were eligible.

## Conclusions

In this study we found that—while individual and community indicators of need increased during the recession—relative contributions of individual predictors as social determinants of health and disparities remained largely consistent over the course of the Great Recession. Young maternal age and low maternal education were the largest magnitude individual predictors of late/no prenatal care utilization during all three recession-related periods. Community and LHD expenditure variables exhibited greater variation—over time, percent voting Republican and 2MCH were both increasingly associated with late/no prenatal care utilization in a non-beneficial direction, while WIC enrollment at the individual level appears to have been protective against late/no prenatal care utilization. These associations should all be further explored. Clustering of individual predictors into low, average, and high social disadvantage scenarios clearly demonstrated the disparate combined probability of late/no entry to prenatal care, as well as persistent racial/ethnic disparity within each level of social advantage/disadvantage. Our findings provide a compelling rationale for targeted outreach to pregnant women with high disadvantage characteristics—particularly those with low education and young age. WIC may represent an effective policy-based approach to reducing disparities in late/no prenatal care utilization and its effects during the Great Recession should be further explored.

## Data Availability

The data that support the findings of this study are available from the Washington State Department of Health and the Florida State Department of Health but restrictions apply to the availability of these data, which were used under license for the current study, and so are not publicly available. Data are however available from the authors upon reasonable request and with permission of the State Departments of Health in Washington and Florida.

## Appendix 1

**Table 7:** Predicted Probability of Late/No PNC by State and Total Study Population for Low, Average, and High Social Status Characteristics

Low Social Disadvantage Case						
	Baseline Period		Period 1		Period 2	
Florida	Prob.* (SE)	[95% Conf. Interval]	Prob.* (SE)	[95% Conf. Interval]	Prob.* (SE)	[95% Conf. Interval]
Non-Hispanic White	0.020* (0.008)	0.005 - 0.035	0.019* (0.009)	0.002 - 0.036	0.026* (0.009)	0.008 - 0.045
Hispanic White	0.039* (0.014)	0.013- 0.066	0.026 (0.015)	-0.003 - 0.055	0.035* (0.013)	0.010 - 0.060
Non-Hispanic Black	0.052* (0.011)	0.031 - 0.074	0.056* (0.013)	0.031 - 0.081	0.063* (0.012)	0.040 - 0.086
Washington						
Non-Hispanic White	0.075* (0.027)	0.022 - 0.129	0.073* (0.024)	0.026 - 0.120	0.073* (0.023)	0.029 - 0.117
Hispanic White	0.095* (0.030)	0.037 - 0.153	0.080* (0.024)	0.033 - 0.127	0.082* (0.023)	0.037 - 0.126
Non-Hispanic Black	0.108* (0.029)	0.051 - 0.165	0.110* (0.024)	0.063 - 0.157	0.110* (0.022)*	0.066 - 0.153
Total						
Non-Hispanic White	0.033* (0.005)	0.024 - 0.043	0.033* (0.006)	0.022 - 0.045	0.039* (0.005)	0.029 - 0.049
Hispanic White	0.053* (0.012)	0.029 - 0.077	0.040* (0.012)	0.017 - 0.063	0.048* (0.009)	0.030 - 0.066
Non-Hispanic Black	0.066* (0.010)	0.048 - 0.084	0.070* (0.010)	0.051 - 0.089	0.076* (0.008)	0.061 - 0.091
Average Case						
	Baseline Period		Period 1		Period 2	
Florida						
Non-Hispanic White	0.103* (0.009)	0.086 - 0.120	0.112* (0.009)	0.095 - 0.129	0.108* (0.009)	0.091 - 0.126
Hispanic White	0.123* (0.014)	0.096 - 0.149	0.119* (0.013)	0.093 - 0.145	0.117* (0.012)	0.093 - 0.141
Non-Hispanic Black	0.135* (0.010)	0.116 - 0.155	0.148* (0.011)*	0.127 - 0.170	0.145* (0.011)	0.124 - 0.166
Washington						
Non-Hispanic White	0.159* (0.027)	0.106 - 0.211	0.166* (0.024)	0.119 - 0.212	0.155* (0.023)	0.110 - 0.200
Hispanic White	0.178* (0.029)	0.121 - 0.235	0.173* (0.023)	0.128 - 0.218	0.164* (0.023)	0.120 - 0.208
Non-Hispanic Black	0.191* (0.028)	0.136 - 0.246	0.203* (0.023)	0.158 - 0.247	0.192* (0.022)	0.148 - 0.235
Total						
Non-Hispanic White	0.116* (0.005)	0.106 - 0.126	0.126* (0.005)	0.116 - 0.136	0.121* (0.005)	0.111 - 0.131
Hispanic White	0.136* (0.012)	0.113 - 0.159	0.133* (0.010)	0.114 - 0.152	0.130* (0.008)	0.113 - 0.146
Non-Hispanic Black	0.149* (0.008)	0.133 - 0.164	0.163* (0.007)	0.149 - 0.178	0.158* (0.007)	0.145 - 0.171
High Social Disadvantage Case						
Florida	Prob.* (SE)	[95% Conf. Interval]	Prob.* (SE)	[95% Conf. Interval]	Prob.* (SE)	[95% Conf. Interval]
Non-Hispanic White	0.432* (0.037)	0.361 - 0.504	0.431* (0.031)	0.371 - 0.491	0.366* (0.023)	0.320 -0.412
Hispanic White	0.452* (0.036)	0.382 - 0.522	0.438* (0.029)	0.381 - 0.495	0.375* (0.024)	0.329 - 0.421
Non-Hispanic Black	0.465* (0.035)	0.396 – 0.533	0.468* (0.028)	0.413 - 0.522	0.403* (0.023)	0.358 - 0.447
Washington						
Non-Hispanic White	0.488* (0.042)	0.406 - 0.569	0.485* (0.039)	0.408 - 0.562	0.413* (0.033)	0.348 -0.477
Hispanic White	0.507* (0.041)	0.427 - 0.588	0.492* (0.036)	0.421 - 0.564	0.421* (0.032)	0.359 – 0.484
Non-Hispanic Black	0.520* (0.041)	0.441 - 0.600	0.522* (0.036)	0.452 - 0.593	0.449* (0.032)	0.387 - 0.512
Total						
Non-Hispanic White	0.446* (0.035)	0.377 - 0.514	0.446* (0.030)	0.386 - 0.505	0.379* (0.023)	0.334 - 0.423
Hispanic White	0.465* (0.034)	0.398 – 0.532	0.452* (0.028)	0.397 - 0.508	0.387* (0.022)	0.344 – 0.431
Non-Hispanic Black	0.478* (0.033)	0.412 - 0.543	0.482* (0.027)	0.429 - 0.535	0.415* (0.022)	0.373 - 0.458

**P* < .05 was used to establish statistical significance and is indicated with an asterisk(*)

## Appendix 2

**Table 8:** Logit Model Results of Late/No PNC in Total Study Population (WA + FL)

	Baseline Period (*n*=270775)			Period 1 (*n*=195921)			Period 2 (*n*=178254)		
	Coef-ficient	Robust SE	95% Conf. Interval	Coeff-icient	Robust SE	95% Conf. Interval	Coef-ficient	Robust SE	95% Conf. Interval
Maternal Race/Ethnicity									
Non-Hispanic White (1)	referent			referent			referent		
Hispanic White (2)	0.0840	0.0554	-0.0245 - 0.1925	0.0090	0.0479	-0.0848 - 0.1028	0.0334	0.0427	-0.0504 - 0.1171
Non-Hispanic Black (3)	0.2247*	0.0365	0 .1532 - 0.2962	0.2509*	0.0403	0.1720 - 0.3298	0.2578*	0.0307	0.1977 - 0.3180
Age									
< 14 years (1)	1.3213*	0.0702	1.1838 - 1.4588	1.3342*	0.0893	1.1592 - 1.5092	1.3447	0.1420*	1.0664 - 1.6230
15-19 years (2)	0.6644*	0.0442	0.5779 - 0.7510	0.6259*	0.0635	0.5015 - 0.7504	0.6003*	0.0382	0.5255 - 0.6751
20-24 years (3)	0.4019*	0.0361	0.3311 - 0.4727	0.4602*	0.0394	0.3831 - 0.5374	0.3830*	0.0256	0.3329 - 0.4331
25-29 years (4)	0.1402*	0.0242	0.0927 - 0.1877	0.1640*	0.0323	0.1007 - 0.2274	0.1581*	0.0232	0.1126 - 0.2036
30-34 years (5)	referent			referent			referent		
35-39 years (6)	0.0371	0.0289	-0.0195 - 0.0937	0.0618*	0.0305	0.0020 - 0.1215	0.1214*	0.0523	0.0190 - 0.2239
40+ years (7)	0.5232*	0.0669	0.3921 - 0.6544	0.4838*	0.0552	0.3757 - 0.5918	0.3337*	0.0764	0.1840 - 0.4835
Marital Status									
Married (1)	referent			referent			referent		
Not Married (0)	0.3268*	0.0206	0.2864 - 0.3673	0.3368*	0.0339	0.2703 - 0.4034	0.2760*	0.0233	0.2303 - 0.3217
Foreign-Born Status									
Not Foreign-Born (0)	referent			referent			referent		
Foreign-Born (1)	0.2781*	0.0675	0.1458 - 0.4105	0.2366*	0.0533	0.1321 - 0.3410	0.2000*	0.0469	0.1080 - 0.2919
Education									
Less than HS education (1)	0.5444*	0.0486	0.4492 - 0.6396	0.4801*	0.0531	0.3750 - 0.5842	0.3942*	0.0494	0.2974 - 0.4910
HS diploma or GED (2)	0.2214*	0.0313	0.1602 - 0.2827	0.1641*	0.0360	0.0935 - 0.2347	0.1924*	0.0269	0.1397 - 0.2451
Some College (3)	referent			referent			referent		
Age <20; ed attainment not assessed (4)	0.3924*	0.0412	0.3116 - 0.4731	0.3712*	0.0439	0.2851 - 0.4572	0.2897*	0.0401	0.2111 - 0.3683
Insurance Payer									
Medicaid (1)	0.7979*	0.0419	0.7157 - 0.8800	0.8669*	0.0486	0.7716 - 0.9621	0.8214*	0.0494	0.7246 - 0.9182
Private Insurance (2)	referent			referent			referent		
Self-Pay/ Uninsured (3)	1.0720*	0.0718	.9313419 1.21263	1.2122*	0.0652	1.0844 - 1.3401	1.0784*	0.0751	0.9311 - 1.2256
Other (Indian Health Service, CHAMPUS, Tricare, etc.) (8)	0.5480*	0.1433	0.2672 - 0.8289	0.5433*	0.1654	0.2192 - 0.8674	0.6496*	0.1162	0.4218 - 0.8774
Unknown (9)	0.4226*	0.1593	0.1104 - 0.7348	0.5182*	0.1979	0.1303 - 0.9061	0.5764*	0.1690	0.2452 - 0.9077
WIC Enrollment Status									
Yes WIC (1)	-0.0564	0.0307	-0.1166 - 0.0039	-0.0463	0.0351	-0.1151 - 0.0224	-0.0363	0.0407	-0.1161 - 0.0435
No WIC (0)	referent			referent			referent		
Unemployment Rate	-0.0121	0.0552	-0.1204 - 0.0962	-0.0032	0.0075	-0.0179 - 0.0116	-0.0061	0.0310	-0.0668 - 0.0546
Community Poverty	-0.2033	0.1094	-0.4177 - 0.0111	-0.2882*	0.1337	-0.5501 -0.0262	-0.3882*	0.1345	-0.6518 -0.1246
Median HH Income	0.0000	0.0000	-0.0000 - 0.0000	0.0000	0.0000	-3.48e-06 - 0.0000	0.0000	0.0000	-9.57e-06 - 0.0000
Core Based Statistical Area									
Metro-politan (1)	referent			referent			referent		
Micro-politan (2)	0.0604	0.1059	-0.1471 - 0.2680	0.0140	0.1182	-0.2176 0.2456	0.1245	0.1051	-0.0816 - 0.3306
Rural (3)	-0.0860	0.1351	-0.3508 - 0.1789	-0.1549	0.1430	-0.4352 - 0.1255	-0.1983	0.1714	-0.5342 - 0.1377
Gini Coefficient	-0.1083	1.6231	-3.2895 - 3.0730	-1.4975	1.7262	-4.8808 - 1.8857	-1.2660	1.7388	-4.6740 - 2.1420
Percent Republican	0.0097*	0.0034	0.0031 - 0.0163	0.0121*	0.0044	0.0035 - 0.0208	0.0140*	0.0048	0.0047 - 0.0234
Per Capita MDs (GPs and FM)	-10.7779*	5.2387	-21.0455 -0.5104	-6.9354	4.6536	-16.0562 - 2.1854	-6.461736	5.1762	-16.6070 - 3.6835
LHD Per Capita 2MCH Expenditures	0.0073	0.0062	-0.0048 - 0.0193	0.0139*	0.0059	0.0023 - 0.0254	0.0190*	0.0061	0.0071 - 0.0309
LHD Per Capita WIC Expenditures	-0.0060	0.0187	-0.0427 - 0.0306	-0.0070	0.0171	-0.0405 - 0.0264	-0.0143	0.0200	-0.0535 - 0.0249
State									
Florida (1)	referent			referent			referent		
Washington (2)	0.4144	0.2325	-0.0413 - 0.8702	0.4335*	0.2076	0.0267 - 0.8404	0.3912	0.2105	-0.0214 - 0.8038
Constant	-3.3989	1.0361	-5.4296 -1.3683	-3.3255*	0.9594	-5.2059 -1.4452	-3.23935	1.2188	-5.6281 - -0.8506

**P* < .05 was used to establish statistical significance and is indicated with an asterisk(*)

## Appendix 3

**Table 9:** WA-only Regression Model Results

Washington State: Late/No PNC									
	Coef.	Robust Std. Err.	95% Conf. Interval	Coef.	Robust Std. Err.	95% Conf. Interval	Coef.	Robust Std. Err.	95% Conf. Interval
	Baseline (n= 64814)			Period 1 (n= 52460)			Period 2 (n= 48628)		
Maternal Race/Ethnicity									
Non-Hispanic White (1)	referent			referent			referent		
Hispanic White (2)	0.038*	0.011	0.016 - 0.061	0.023	0.012	-0.002 - 0.047	0.030*	0.007	0.017 - 0.044
Non-Hispanic Black (3)	-0.013*	0.012	-0.038 - 0.013	0.015	0.008	-0.001 - 0.032	0.006	0.007	-0.008 - 0.021
Age									
< 14 years (1)	0.298*	0.032	0.233 - 0.364	0.341*	0.057	0.226 - 0.457	0.306*	0.069	0.166 - 0.446
15-19 years (2)	0.147*	0.011	0.124 - 0.169	0.144*	0.014	0.116 - 0.172	0.113*	0.007	0.099 - 0.126
20-24 years (3)	0.062*	0.006	0.049 - 0.075	0.078*	0.008	0.061 - 0.095	0.056*	0.005	0.045 - 0.066
25-29 years (4)	0.014*	0.004	0.007 - 0.021	0.021*	0.005	0.011 - 0.031	0.017*	0.002	0.012 - 0.022
30-34 years (5)	referent			referent			referent		
35-39 years (6)	0.000	0.003	-0.006 - 0.007	0.005	0.004	-0.003 - 0.014	0.007	0.008	-0.010 - 0.023
40+ years (7)	0.002	0.005	-0.010 - 0.013	0.039*	0.009	0.020 - 0.057	0.013	0.009	-0.006 - 0.032
Marital Status									
Married (1)	referent			referent			referent		
Not Married (0)	0.059*	0.008	0.042 - 0.077	0.053*	0.014	0.025 - 0.082	0.045*	0.008	0.028 - 0.061
Foreign-Born Status									
Not Foreign-Born (0)	referent			referent			referent		
Foreign-Born (1)	0.056*	0.010	0.035 - 0.076	0.066*	0.011	0.043 - 0.089	0.063*	0.007	0.048 - 0.078
Education									
Less than HS education (1)	0.058*	0.015	0.042-0.077	0.051*	0.008	0.034 - 0.067	0.037*	0.012	0.012 - 0.061
HS diploma or GED (2)	0.022*	0.006	0.011-0.034	0.010	0.006	-0.002 - 0.021	0.026*	0.006	0.014 - 0.038
Some College (3)	referent			referent			referent		
Insurance Payer									
Medicaid (1)	0.089*	0.011	0.067 - 0.110	0.101*	0.016	0.068 - 0.134	0.084*	0.012	0.060 - 0.108
Private Insurance (2)	referent			referent			referent		
Self-Pay/ Uninsured (3)	0.187*	0.015	0.157 - 0.216	0.224*	0.024	0.175 - 0.273	0.153*	0.023	0.107 - 0.198
Other (Indian Health Service, CHAMPUS, Tricare, etc.) (8)	0.030	0.026	-0.023 - 0.083	0.022	0.029	-0.037 - 0.081	0.047*	0.021	0.005 - 0.090
Unknown (9)	0.010	0.019	-0.029 - 0.049	0.047	0.040	-0.035 - 0.129	0.041	0.027	-0.013 - 0.095
WIC Enrollment Status									
Yes WIC (1)	-0.004	0.007	-0.019 - 0.011	0.006	0.010	-0.014 - 0.027	0.003	0.013	-0.023 - 0.030
No WIC (0)	referent			referent			referent		
Unemployment Rate	-0.002	0.009	-0.020 - 0.015	0.000	0.002	-0.003 - 0.003	-0.005	0.005	-0.015 - 0.005
Community Poverty	-0.019	0.024	-0.068 - 0.030	-0.061*	0.030	-0.123 - 0.001	-0.081*	0.027	-0.136 - -0.026
Median HH Income	2.35E-06	2.48E-06	-2.69e-06 - 7.40e-06	4.71E-06	3.03E-06	-1.45e-06 - 0.000	1.79E-06	2.47E-06	-3.22e-06 - 6.81e-06
Core Based Statistical Area									
Metro-politan (1)	referent			referent			referent		
Micro-politan (2)	0.027	0.025	-0.024 - 0.078	0.020	0.029	-0.039 - 0.040	0.018	0.027	-0.037 - 0.073
Rural (3)	0.061	0.043	-0.027 - 0.149	0.089*	0.042	0.004 - 0.175	0.059	0.042	-0.026 - 0.145
Gini Coefficient	0.251	0.530	-0.826 - 1.328	0.670	0.572	-0.491 - 1.831	-0.300	0.465	-1.245 - 0.646
Percent Republican	-0.0000	0.0017	-0.0035 - 0.0034	0.0016	0.0027	-0.0039 - 0.0072	0.0033	0.0021	-0.0010 - 0.0075
Per Capita MDs (GPs and FM)	-2.053*	0.603	-3.278 - -0.828	-1.449*	0.650	-2.770 – -0.128	-1.157	0.702	-2.584 - 0.269
LHD Per Capita 2MCH Expenditures	-0.0011	0.0025	-0.0062 - 0.0040	-0.0010	0.0030	-0.0070 - 0.0051	0.0042	0.0022	-0.0002 - 0.0086
LHD Per Capita WIC Expenditures	-0.0019	0.0067	-0.0156 - 0.0118	-0.0043	0.0074	-0.0193 - 0.0106	-0.0088	0.0058	-0.0207 - 0.0030
Constant	-0.0750	0.3464	-0.7789 0.6290	-0.4806	0.4286	-1.3517 - 0.3904	0.0392	0.3394	-0.6506 - 0.7290

**P* < .05 was used to establish statistical significance and is indicated with an asterisk(*)

## Appendix 4

**Table 10:** FL-only Regression Model Results

Florida: Late/No PNC									
	Coef.	Robust Std. Err.	95% Conf. Interval	Coef.	Robust Std. Err.	95% Conf. Interval	Coef.	Robust Std. Err.	95% Conf. Interval
	Baseline n= 205961			Per 1 n=143461			Per 2 n=129626		
Maternal Race/Ethnicity									
Non-Hispanic White (1)	referent			referent			referent		
Hispanic White (2)	0.0142	0.0111	-0.0080 - 0.0364	0.0006	0.0095	-0.0184 - 0.0195	0.0007	0.0077	-0.0146 - 0.0161
Non-Hispanic Black (3)	0.0362*	0.0058	0.0245 - 0.0478	0.0375*	0.0063	0.0250 - 0.0500	0.0348*	0.0050	0.0248 - 0.0447
Age									
< 14 years (1)	0.2522*	0.0159	0.2204 - 0.284	0.2431*	0.0187	0.2057 - 0.2804	0.2512*	0.0344	0.1825 - 0.3120
15-19 years (2)	0.0899*	0.0053	0.0793 - 0.1005	0.0792*	0.0084	0.0626 - 0.0959	0.0816*	0.0052	0.0713 - 0.0919
20-24 years (3)	0.0306*	0.0029	0.0250 - 0.0363	0.0360*	0.0026	0.0307 - 0.0412	0.0310*	0.0037	0.0236 - 0.0383
25-29 years (4)	0.0070*	0.0021	0.0027 - 0.0113	0.0074*	0.0031	0.0012 - 0.0136	0.0086*	0.0031	0.0025 - 0.0147
30-34 years (5)	referent				referent				
35-39 years (6)	0.0033	0.0030	-0.0026 - 0.0093	0.0043*	0.0032	-0.0020 - 0.0106	0.0108*	0.0048	0.0013 - 0.0204
40+ years (7)	0.0586*	0.0091	0.0404 - 0.0768	0.0449*	0.0082	0.0286 - 0.0612	0.0336*	0.0086	0.0164 - 0.0507
Marital Status									
Married (1)	referent			referent			referent		
Not Married (0)	0.0373*	0.0023	0.0328 - 0.0419	0.0393*	0.0052	0.0289 - 0.0497	0.0288*	0.0037	0.0214 - 0.0362
Foreign-Born Status									
Not Foreign-Born (0)	referent			referent			referent		
Foreign-Born (1)	0.0333*	0.0112	0.0110 - 0.0557	0.0217*	0.0078	0.0061 - 0.0372	0.0148*	0.0058	0.0031 - 0.0264
Education									
Less than HS education (1)	0.0918*	0.0118	0.0683 - 0.1153	0.0896*	0.0138	0.0621 - 0.1170	0.0701*	0.0079	0.0543 - 0.0860
HS diploma or GED (2)	0.0217*	0.0053	0.0111 - 0.0323	0.0193*	0.0067	0.0059 - 0.0327	0.0200*	0.0052	0.0096 - 0.0305
Some College (3)	referent			referent			referent		
Age <20; ed attainment not assessed (4)	0.0645*	0.0072	0.0502 - 0.0788	0.0656*	0.0086	0.0484 - 0.0829	0.0464*	0.0075	0.0315 - 0.0614
Insurance Payer									
Medicaid (1)	0.1041*	0.0077	0.0887 - 0.1196	0.1156	0.0091	0.0975 - 0.1337	0.1033*	0.0088	0.0857 - 0.1210
Private Insurance (2)	referent			referent			referent		
Self-Pay/ Uninsured (3)	0.1561*	0.0207	0.1148 - 0.1973	0.1757	0.0170	0.1418 - 0.2096	0.1432*	0.0190	0.1053 - 0.1810
Other (Indian Health Service, CHAMPUS, Tricare, etc.) (8)	0.0752*	0.0304	0.0144 - 0.1360	0.0840	0.0351	0.0141 - 0.1540	0.0814*	0.0235	0.0345 - 0.1284
Unknown (9)	0.0959*	0.0306	0.0349 - 0.1569	0.0654	0.0274	0.0107 - 0.1202	0.0813*	0.0350	0.0113 - 0.1512
WIC Enrollment Status									
Yes WIC (1)	-0.0156*	0.0052	-0.0261 -0.0051	-0.0176*	0.0054	-0.0283 - -0.0069	-0.0133*	0.0057	-0.0246 -0.0020
No WIC (0)	referent			referent			referent		
Unemployment Rate	-0.0053	0.0097	-0.0247 - 0.0140	-0.0009	0.0010	-0.0029 - 0.0011	-0.0009	0.0055	-0.0119 - 0.0101
Community Poverty	-0.0417	0.0277	-0.0971 - 0.0137	-0.0339	0.0279	-0.0896 - 0.0218	-0.0451	0.0256	-0.0961 - 0.0060
Median HH Income	0.0000	0.0000	-2.69e-06 - 4.02e-06	0.0000	0.0000	-1.66e-06 - 4.30e-06	0.0000	0.0000	-2.57e-06 3.84e-06
Core Based Statistical Area									
Metro-politan (1)	referent			referent			referent		
Micro-politan (2)	0.0140	0.0229	-0.0318 - 0.0597	0.0046	0.0290	-0.0532 - 0.0624	0.0288	0.0244	-0.0200 - 0.0775
Rural (3)	-0.0290	0.0282	-0.0853 - 0.0272	-0.0534	0.0299	-0.1130 - 0.0063	-0.0537	0.0394	-0.1323 - 0.0249
Gini Coefficient	0.1556	0.2475	-0.3386 - 0.6498	-0.1628	0.2699	-0.7017 - 0.3762	-0.0573	0.2550	-0.5664 - 0.4517
Percent Republican	0.0013	0.0007	-0.0002 - 0.0028	0.0016*	0.0007	0.0001 - .0030864	0.0018*	0.0008	0.0002 - 0.0033
Per Capita MDs (GPs and FM)	-1.4921	0.9801	-3.449 - .4648048	-1.0235	0.8486	-2.7178 - 0.6709	-0.7734	1.0032	-2.7764 - 1.2296
LHD Per Capita 2MCH Expenditures	0.0019	0.0016	-0.0013 - 0.0051	0.0022	0.0013	-0.0005 - 0.0048	0.0028*	0.0013	0.0001 - 0.0055
LHD Per Capita WIC Expenditures	0.0002	0.0037	-0.0071 - 0.0075	-0.0009	0.0030	-0.0069 - 0.0052	-0.0013	0.0032	-0.0077 - 0.0052
Constant	-0.0979	0.1822	-0.4618 - 0.2659	-0.0125	0.1727	-0.3572 - 0.3324	-0.0337	0.2047	-0.4424 - 0.3751

**P* < .05 was used to establish statistical significance and is indicated with an asterisk(*)

## Abbreviations

2MCH: combined FP and MICA expenditures
ACA: Affordable Care Act
AIC: Akaike information criteria
ARRA: American Recovery and Reinvestment Act
BIC: Bayesian information criteria
BW/LBW: Birth weight/Low birth weight
CBSA: Core-based statistical area
CHAMPUS: Civilian Health and Medical Program of the Uniform Services
CONSORT: Consolidated Standards of Reporting Trials
DFB: Difference from best
DHHS: Department of Health and Human Services
DOH: Department of Health
FL: State of Florida
FM: Family medicine
FP: Family planning
FY: Fiscal year
GA: Gestational age
GED: General education diploma
GP: General practitioner
GR: Great Recession (December 2007–June 2009)
HP: Healthy People
HS: High school
IID: Increase in disparity
IM: Infant mortality
IRB: Institutional Review Board
LBW: Low birth weight (< 2500 g)
LHD: Local health department
LHJ: Local health jurisdiction
LPM: Linear probability (regression) model
MCH: Maternal/Child health
MD: Medical doctor
MICA: Maternal, infant, child, and adolescent (service line composite LHD expenditures)
MICH: Maternal, infant, and child health
NBER: National Bureau of Economic Research
PHAST: Public Health Activities and Services Tracking Study
PNC: Prenatal care
PTB32: Preterm birth < 32 weeks
PTB37: Preterm birth < 37 weeks
SD: Standard deviation
SES: Socioeconomic status
SNAP: Supplemental Nutrition Assistance Program
TANF: Temporary Aid for Needy Families
U.S.: United States
USDA: United States Department of Agriculture
VLBW: Very low birth weight (< 1500 g)
WA: State of Washington
WIC: Special Supplemental Nutrition Program for Women, Infants, and Children
